# Evolutionary Medicine: Why do humans get bunions?

**DOI:** 10.1093/emph/eox001

**Published:** 2017-02-11

**Authors:** Pierre Tamer, Scott Simpson

**Affiliations:** 1 Department of Anatomy, School of Medicine, Case Western Reserve University, Cleveland, OH, USA; 2 Department of Anatomy, Case Western Reserve University, Cleveland, OH, USA

## HALLUX VALGUS

### Targeted pathology

Hallux valgus is the most common forefoot problem in adults. It is characterized by an anatomical deformity involving lateral deviation of the hallucial phalanges and medial prominence of the first metatarsal head, commonly known as a bunion. This acquired deformity is often correlated with foot pain, imbalance and impaired gait, falls in older adults and decreased health-related quality of life. Epidemiological studies report a higher prevalence of hallux valgus with increasing age and in more females than males, consistent with potential causative factors such as osteoarthritis and narrowed footwear [1]. Although footwear can be a significant external contributing factor, we propose there are intrinsic and evolutionarily based aspects of the human foot which increase this risk. 

## EVOLUTIONARY PERSPECTIVES

**Figure 1.**
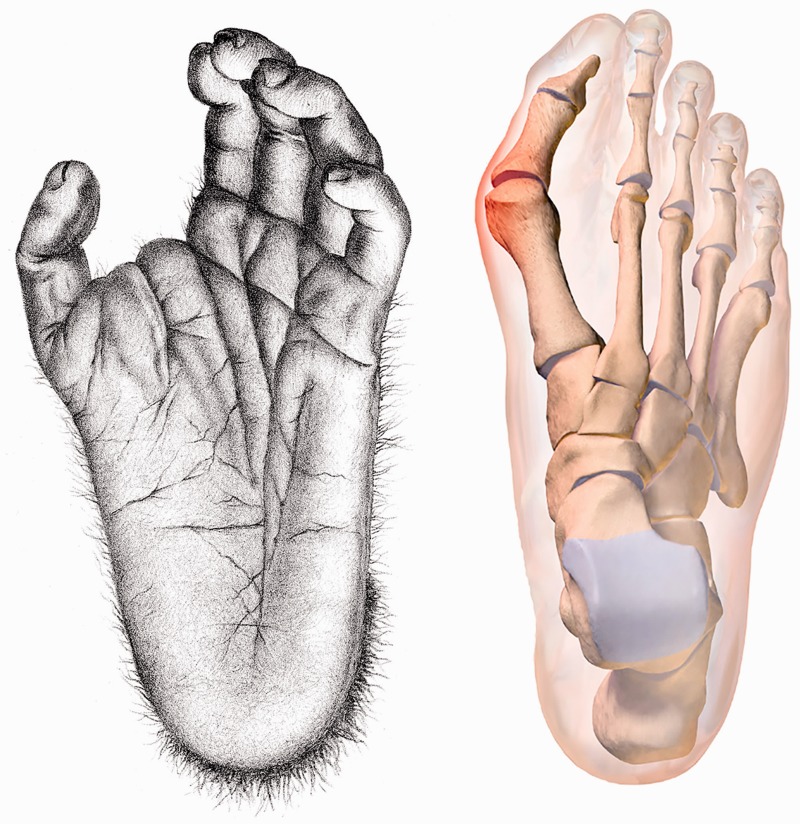
Grasping primate foot with abducent hallux (left); hallux valgus in humans (right) Note the orientation of the plantar surface in the chimpanzee’s abducent hallux

**Figure 2.**
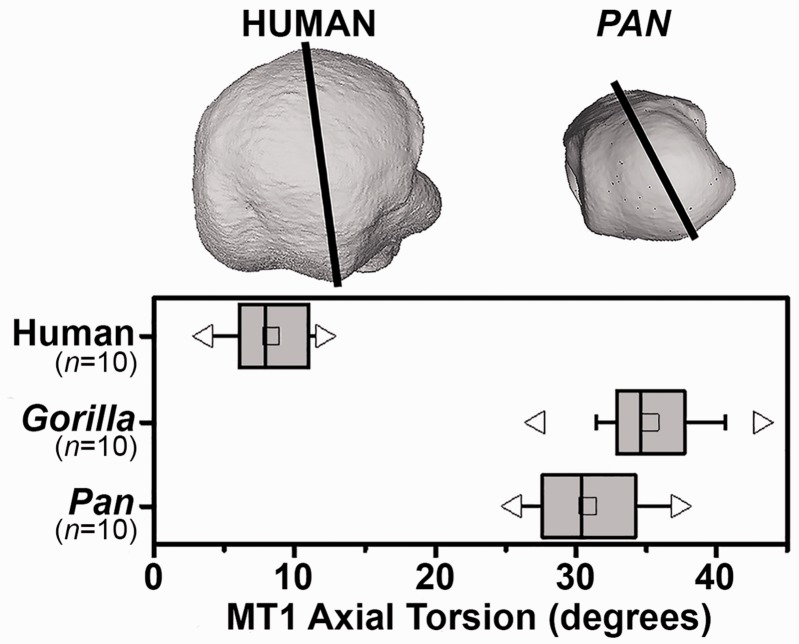
Axial torsion of first metatarsal heads in humans, gorillas and chimpanzees normalized by the basal dorso-plantar axis. Medial is to the left in upper image

The human foot differs from that of all other primates in that we use our foot as a rigid lever during the propulsive phase of locomotion rather than as a grasping organ for arboreal climbing (Fig. 1). To facilitate pedal grasping in apes, the hallucial metatarsal exhibits extorsion along its long axis and the lateral metatarsals are internally rotated, which is contrasted with the realignment of the non-abducent human first metatarsal, allowing the plantar surface to be fully in contact with the ground (Fig. 2). The abducted hallux in primates permits an axis of flexion of the hallux orthogonal to the lateral phalanges, while in humans the adducted and rotated hallux creates an axis of flexion in the same plane as the lateral phalanges, facilitating toe-off at the metatarsophalangeal joint [2]. Primarily as a consequence of hallucial torsion, when the hallucial phalangeal flexors—Flexor Hallucis Longus and Flexor Hallucis Brevis—contract during propulsion, their course shears obliquely across the inferior aspect of the metatarsal head. Although this orientation is normally balanced by constitutive ligaments and muscles, under increasing stress the oblique orientation of the phalangeal flexors can laterally discplace the hallucial sesamoids and the intervening Flexor Hallucis Longus tendon, thereby deflecting the hallucial phalanges laterally.

## FUTURE IMPLICATIONS

Further addressing the evolutionary based anatomy and mechanisms underlying the propensity of humans to develop hallux valgus promotes a better understanding of the frequency of its occurrence, behavioral changes to reduce risk and the development of future therapies. Currently, therapies vary by the severity of the deformity, ranging from non-operative medications and inlays under the forefoot to surgical procedures such as osteotomies or arthrodesis. Within all of these procedures, however, challenges remain, clinical indications vary and high-level evidence is scarce regarding when to use specific operative techniques over others [3]. 

## SUPPLEMENTARY DATA

Supplementary data is available at *EMPH* online.

## Supplementary Material

Supplementary Data
