# Supplementary material for: Evolutionary Medicine: Why do humans get bunions?
Source: Evol Med Public Health. 2017 Feb 11;2017(1):48–9. doi: 10.1093/emph/eox001 (PMC5381353; doi:10.1093/emph/eox001)
Supplement: Supplementary Data [file eox001_Supp.pdf]

Pierre Tamer
Case Western Reserve University
School of Medicine
2109 Adelbert Road
Cleveland, OH USA 44106
661-706-1619
pxt161@case.edu

Dr. Scott Simpson
Case Western Reserve University
Department of Anatomy
2109 Adelbert Road
Cleveland, OH USA 44106

Supplementary Methodology and Data

Methodology

Axial torsion of the first metatarsal head relative to its base was measured in a sample of 30 humans, chimpanzees (*Pan troglodytes*), and gorillas (*Gorilla gorilla*) from collections housed at the Hamann-Todd Osteological Collection, Laboratory of Physical Anthropology, Cleveland Museum of Natural History, Cleveland, OH, USA. Specimens were measured with the same dorsal-planar axis of the metatarsal base. All specimens are males.

These measurements reveal a significant reorganization of the hallucial orientation in humans relative to the African apes that reflects our adaptation to plantigrade terrestrial bipediality.

Measurements and Data

Table 1: *Axial Torsion (degrees) of Metatarsal Head*

**Specimen Number**	**Human**		**Specimen Number**	**Gorilla**		**Specimen Number**	**Chimpanzee**
	Axial Torsion			Axial Torsion			Axial Torsion
754	3.68		1407	37.41		1056	32.32
775	6.06		1409	43.06		1708	29.58
815	5.93		1430	40.63		1718	27.62
1493	8.2		1733	37.69		1722	27.18
1520	9.62		1994	33.03		1739	34.21
1522	11.42		2739	31.44		1745	25.61
1542	11.99		2741	36.02		1758	31.16
1556	11.02		2767	33.18		2746	37.265
1574	7.45		3404	27.28		3537	35.57
1577	7.59		3556	32.91		3552	27.71
Average:	8.296		Average:	35.265		Average:	30.8225
Standard Deviation:	2.701987746		Standard Deviation:	4.641315067		Standard Deviation:	3.941257026
Variance:	7.300737778		Variance:	21.54180556		Variance:	15.53350694

Human vs. Gorilla

t-Test: Two-Sample Assuming Unequal Variances

	3.68	*37.41*
Mean	8.808888889	35.0266667
Variance	5.253961111	23.5955
Observations	9	9
Hypothesized Mean Difference	0	
df	11	
t Stat	-14.64361426	
P(T<=t) one-tail	7.33766E-09	
t Critical one-tail	1.795884819	
P(T<=t) two-tail	1.46753E-08	
t Critical two-tail	2.20098516	

Human vs Chimpanzee

t-Test: Two-Sample Assuming Unequal Variances

	3.68	*32.32*
Mean	8.80888889	30.65611111
Variance	5.25396111	17.16373611
Observations	9	9
Hypothesized Mean Difference	0	
df	12	
t Stat	-13.842737	
P(T<=t) one-tail	4.8495E-09	
t Critical one-tail	1.78228756	
P(T<=t) two-tail	9.6989E-09	
t Critical two-tail	2.17881283	
